# Comparative immune responses of pups following modified live virus vaccinations against canine parvovirus

**DOI:** 10.14202/vetworld.2019.1422-1427

**Published:** 2019-09

**Authors:** Jayalakshmi Vasu, Mouttou Vivek Srinivas, Prabhakar Xavier Antony, Jacob Thanislass, Vijayalakshmi Padmanaban, Hirak Kumar Mukhopadhyay

**Affiliations:** 1 Department of Veterinary Microbiology, Rajiv Gandhi Institute of Veterinary Education and Research, Puducherry, India; 2 Department of Veterinary Biochemistry, Rajiv Gandhi Institute of Veterinary Education and Research, Puducherry, India; 3 Department of Veterinary Medicine, Rajiv Gandhi Institute of Veterinary Education and Research, Puducherry, India

**Keywords:** canine parvovirus, hemagglutination inhibition test, serum neutralization test, vaccine immune responses

## Abstract

**Background and Aim:**

Canine parvovirus (CPV) is the most important viral cause of enteritis and mortality in pups. Evaluation and monitoring of pre- and post-vaccine immune responses may help to determine the efficacy of the current vaccination schedule being followed in pups in India. This study aimed to evaluate and monitor the pre- and post-vaccine immune responses of CPV vaccinated pups using hemagglutination inhibition (HI) assay. The neutralizing antibody titer levels were also detected using serum neutralization test (SNT).

**Materials and Methods:**

The pups were categorized into two groups, the double booster and the single booster groups. In this study, serum samples were subjected to HI and SNT for measuring the CPV antibody titer at frequent intervals for up to 6 months from 27 healthy pups following primary and booster CPV vaccinations.

**Results:**

The antibody titers in double booster pups reached their peaks at the 21^st^ day after the second booster vaccination with a geometric mean (GM) of 3.57. The antibody titers in single booster pups reached their peaks at the 21^st^ day after the first booster vaccination with a lower GM of 3.18.

**Conclusion:**

The double booster pups maintained a higher immune response throughout the period of the study compared to single booster pups though the difference in titers was not statistically significant. SNT results indicated that the raised antibody titer was also able to yield virus-neutralizing antibodies. No interfering maternally derived antibodies were found in the pups at the age of primary vaccination (45^th^ day) in our study. Therefore, the second booster vaccination may be useful in maintaining the protective titer for a prolonged period.

## Introduction

Canine parvovirus (CPV) is an important pathogenic virus and is the causative agent of acute hemorrhagic enteritis and myocarditis in dogs. It is highly contagious and often a fatal disease. CPV-2 was first recognized in 1977 and since then, it has been well established as an enteric pathogen of dogs throughout the world with high morbidity (100%) and frequent mortality up to 10% [1]. After the emergence of CPV in the late 1970s [1,2], due to rapid evolution, new antigenic types were evolved as CPV-2a, CPV-2b (CPV2a with Asn426Asp), and CPV-2c (CPV2a with Asn426Glu) and have completely replaced the original CPV-2 [3]. At present, the original CPV-2 is not found in dog population but present only in vaccine formulations [3,4]. Commonly, CPV causes disease in unvaccinated 1-6-month-old pups. Vaccinated pups are usually protected from the disease and from infection, unless immunization fails due to the presence of high titers of maternally derived antibodies (MDAs) [5,6]. The pups with MDA titers ≥1:20 may fail to be immunized successfully and remain susceptible to infection. Maternally derived hemagglutination inhibition (HI) antibody titers ≥1:80 are believed to protect pups from infection, while titers between 1:20 and 1:80 are too low to provide protection, but high enough to prevent active immunization [7]. Earlier, one CPV-2b based vaccine was available in India, but now only CPV-2 based vaccines are widely used for immunoprophylaxis of pups. Recent studies showed that the CPV strains isolated from the field are clustering away from the vaccine strains [8]. Moreover, an increasing proportion of CPV vaccinated dogs was found susceptible to infection in India raising serious concern about the efficacy of the vaccine strains and vaccination process [9].

Most puppies in many countries are first vaccinated with a multivalent vaccine against CPV, canine distemper virus, canine adenovirus type 2, canine parainfluenza virus, and leptospirosis when they are between 6 and 8 weeks of age, with booster vaccinations being given every 3-4 weeks until the age of 16 weeks and possibly 24 weeks in high-risk breeds. All the dogs should receive a booster 1 year after completion of the initial series followed by booster every 3 years [10]. It should be a common practice to avoid direct or indirect contact with potential sources of infection such as other dogs until 1–2 weeks after completion of this course of vaccination.

Additional inoculation of adult dogs with these vaccines is performed annually or as required by the veterinarians’ experience, the rearing environment and the owner’s wish in the hope of a booster effect in the animal’s antibody titer [11]. In addition, some owners and veterinarians erroneously hold the view that infectious diseases such as parvovirus infection can be controlled by frequent vaccination alone [12] and perceive all vaccination programs to be protective for puppies under all circumstances.

Concerns regarding the influence of MDA have also prompted general opposition by practitioners to vaccination at early ages. Both high-titer CPV vaccines and intranasal vaccine administration proved to be good strategies to overcome the problem of MDA interference [7,13]. However, evaluation of the MDA levels in pups would be a more precise approach, establishing the appropriate time for vaccination on the basis of the actual immune status of the animals, rather than relying on a standardized schedule [14]. Evaluation and monitoring of post-vaccine immune responses may also help to determine the efficacy of the current vaccination schedule being followed in pups in India.

This study aimed to evaluate and monitor the pre- and post-vaccine immune responses of CPV vaccinated pups using HI assay. The neutralizing antibody titer levels were also detected using serum neutralization test (SNT).

## Materials and Methods

### Ethical approval

Ethical approval was not necessary for this study. However, samples were collected as per standard collection procedure without harming or giving stress to the animals.

### Collection of serum samples

The pups were categorized into two groups, the single booster (11) and double booster (16) group (Table-1). In the single booster group, the pups were given the primary vaccine (42-45-day-old pups) followed by a single booster dose (after 21 days), whereas in the double booster pups in addition to the above vaccination, another booster dose was given (21 days after the first booster) (Table-1). A commercial multivalent vaccine (containing CPV-2 strain) was used for vaccinating the pups.

**Table 1:** Details of the puppies under the study.

Dog	Breed	Sex	Dam’s vaccination status
Single booster group			
Dog 1	Labrador retriever	Male	Unknown
Dog 2	Doberman	Female	Vaccinated regularly
Dog 3	Doberman	Male	Vaccinated regularly
Dog 4	Labrador retriever	Male	Vaccinated regularly
Dog 5	Mongrel	Male	Unknown
Dog 6	Doberman	Male	Unknown
Dog 7	GSD	Female	Unknown
Dog 8	Rajapalayam	Male	Unknown
Dog 9	Chippipaarai	Male	Unknown
Dog 10	Labrador retriever	Male	Unknown
Dog 11	Mongrel	Female	Unknown

**Double booster group**			

Dog 12	Mongrel	Male	Unknown
Dog 13	GSD	Male	Vaccinated regularly
Dog 14	GSD	Female	Vaccinated regularly
Dog 15	Labrador retriever	Female	Unknown
Dog 16	Mongrel	Male	Unknown
Dog 17	Mongrel	Female	Vaccinated regularly
Dog 18	GSD	Male	Vaccinated regularly
Dog 19	Dalmatian	Male	Vaccinated regularly
Dog 20	Mongrel	Male	Unknown
Dog 21	Rajapalayam	Male	Vaccinated regularly
Dog 22	GSD	Male	Vaccinated regularly
Dog 23	Mongrel	Female	Vaccinated regularly
Dog 24	Mongrel	Male	Unknown
Dog 25	Alsatian	Female	Unknown
Dog 26	Alsatian	Female	Unknown
Dog 27	Mongrel	Male	Unknown

Blood samples were collected from all the vaccinated pups on the day of primary CPV vaccination (considered as 0 day), followed by the 21^st^ day (first booster vaccination), 42^nd^ day (second booster vaccination), 63^rd^ day, 84^th^ day, 105^th^ day, 126^th^ day, 147^th^ day, and 168^th^ day post-primary vaccination. The collected blood samples were allowed to clot. Then, the tubes were centrifuged and the serum was collected and stored at −20°C until further testing.

### HI test

Two-fold serial dilutions of 25 µl of serum samples were made in 25 µl of 0.2 M Sorenson’s phosphate-buffered saline (PBS) (pH 7.0) followed by the addition of 4 HA units of CPV-2 vaccine strain. A commercial vaccine containing CPV-2 strain (>10^7.0^ TCID_50_/dose) was used for preparing 4 HAU. The plates were incubated at 37°C for 45 min followed by 4°C for 30 min. Then, 50 µl of 0.65% of pig erythrocytes were added with gentle mixing and left at 4°C for 1 h. Cell controls in the form of 50 µl of 0.2 M Sorenson’s PBS and 50 µl of 0.65% of pig erythrocytes and virus controls with 25 µl of 0.2 M Sorenson’s PBS, 25 µl of 4 HA units of virus, and 50 µl of 0.65% of pig erythrocytes and serum controls with 25 µl of 0.2 M Sorenson’s PBS, 25 µl of serum samples, and 50 µl of 0.65% of pig erythrocytes were also kept [15]. The HI titer was expressed as the reciprocal of the highest dilution of the serum that completely inhibited the HA activity. The log_10_ geometric mean (GM) was calculated by converting the HI titers into log_10_ values (X) and using the formula 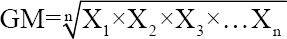, where n = total number of pups. The data analysis was performed using the MS Office Excel 2007 software.

### SNT

Fifty microliter of chilled DMEM was added to the wells labeled as A1-A12. Using a sterile tip, 50 µl of test serum was added to the first well (A1), thus making a serum dilution to 1:2 and mixed well by pipetting. Serial two-fold dilutions of test serum were made from A1 to A12. Wells were mixed after each transfer and continued dilution until the end. After the final dilution, 50 µl was discarded. Using a sterile tip, an equal volume (50 µl) containing 100 TCID_50_ of the virus (CPV-2a strain maintained in the Department of VMC, RIVER, Puducherry) was added to each well of the serum dilution. The plates were incubated at 37°C for 1 h. After incubation, 100 µl of a suspension containing 20,000 CRFK cells were added to the above virus-serum mixture. The cells were distributed evenly by rocking the plate back and forth and from side to side. Then, the plates were incubated at 37°C for 5 days in 3.5% humidified CO_2_ incubator. Following incubation, the plates were subjected to three freeze-thaw cycles. The undiluted cryolysate was centrifuged at 6000 rpm at 4°C for 10 min, and the supernatants were tested for CPV hemagglutination activity using freshly prepared 0.65% of pig erythrocytes [16].

Neutralizing antibody titers were calculated as the reciprocal of the highest serum dilution that completely neutralized the virus (absence of HA activity). The log_10_ GM was calculated by converting the SNT titers into log_10_ values. The statistical analysis was performed using the t-test. p≤0.05 was considered statistically significant. The data analysis was performed using the MS Office Excel 2007 software (Microsoft, USA).

## Results and Discussion

### HI test

**Figure-1:**
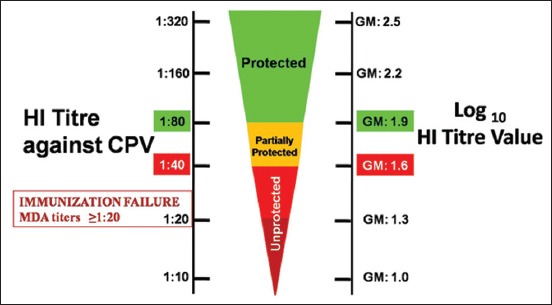
Hemagglutination inhibition (HI) titer (left side) and log10 HI titer scale (right side) to determine the protection, partial protection, and unprotected pups against canine parvovirus.

No interfering MDA was detected by HI test during primary vaccination (0 day), i.e., at the age of the 45^th^ day of the pups. However, at the same time, the pups were fully susceptible to CPV infection. This may be due to poor adherence of the vaccination schedule to be followed in dams by the canine breeders/owners in India in the absence of any strict regulations/guidelines. According to researchers such as Carmichael [5], Pratelli *et al*. [16], and Tagushi *et al*. [17], a HI titer of ≥1:80 (equivalent to ≥1.9 GM log_10_ HI titer) was enough to protect the pups against virulent challenge, whereas pups having HI titer of <1:40 (<1.6 GM log_10_ HI titer) were fully susceptible (Figure-1). The pups having titer between 1:40 and 1:80 (1.6-1.9 GM log_10_ HI titer) were considered partially protected. In another study, Carmichael [5] observed that the pups with MDA titers ≥1:20 (equivalent to >1.3 GM log_10_ HI titer) may fail to be immunized successfully and remain susceptible to infection.

**Figure-2:**
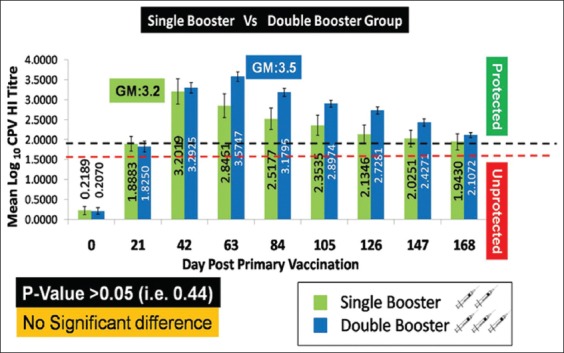
Determination of canine parvovirus antibody titer by hemagglutination inhibition (HI) assay. The value indicates geometric mean log10 HI titer ± standard error.

In the present study, the GM log_10_ HI titers of serum samples at various days post-immunization from the single booster and double booster pups are depicted in Table-2 and Figure-2. The antibody titers of the pups raised substantially almost reaching the protective level (≥1:80/>1.9 GM log_10_ HI titer) after primary vaccination in both the single and the double booster vaccinated pups. In the double booster group, the pups showed the peak HI titer (GM: 3.57) at the 63^rd^ day post-primary vaccination, i.e., 21^st^ day after the second booster vaccination followed by a gradual decrease in antibody titer until the end of the study period (168^th^ day post-primary vaccination). The pups in the double booster group were also able to maintain the protective HI titers until the end of the study period (GM: 2.10). The pups in the single booster group, on the other hand, reached their peak HI tires (GM: 3.20) at the 42^nd^ day post-primary vaccination, i.e., 21^st^ day after the first booster vaccination, which was slightly lower when compared to the double booster group. The pups in the single booster group were also able to maintain the protective titer at the end of the study period (GM: 1.94) though the titers were found to be slightly lower than the double booster group. Although the difference in HI titers between the two groups of the pups was not statistically significant (p>0.05, i.e., 0.44), the pups under the double booster group maintained a better and higher immune response than the single booster group. Therefore, it was inferred from this study that the second booster vaccination will be extremely useful to attain a higher antibody titer for a prolonged period.

**Table 2:** Geometric mean log10 antibody titers of immunized pups against canine parvovirus at different days post-vaccination.

Test	Group	Day post-primary vaccination								

		0	21	42	63	84	105	126	147	168
Hemagglutination inhibition test	Single booster pups	0.21±0.10	1.88±0.19	3.20±0.33*	2.84±0.30	2.51±0.27	2.35±0.25	2.13±0.23	2.02±0.21	1.94±0.20
	Double booster pups	0.20±0.08	1.82±0.14	3.29±0.13	3.57±0.12*	3.17±0.10	2.89±0.09	2.72±0.09	2.42±0.09	2.10±0.06
Serum neutralization test (protective titer)	Single booster pups	0.37±0.23	2.25±0.09	3.46±0.26*	3.08±0.19	2.70±0.17	2.40±0.12	2.33±0.14	2.18±0.14	1.95±0.09
	Double booster pups	0.67±0.23	1.95±0.19	3.68±0.14	3.83±0.14*	3.38±0.26	3.01±0.21	2.70±0.17	2.63±0.23	2.33±0.14

The value indicates geometric mean log_10_ antibody titer±standard error.

* Indicates peak GM log_10_ antibody titer value

### SNT

**Figure-3:**
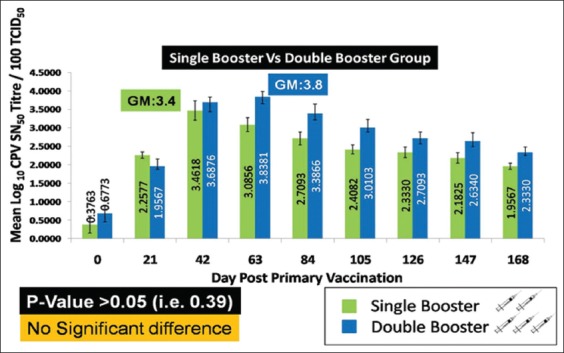
Determination of canine parvovirus-neutralizing antibody titer by serum neutralization test (SNT). The value indicates geometric mean log10 SNT titer ± standard error.

The log_10_ GM neutralizing antibody titers, the single and double booster pups at different days post-vaccination, are displayed in Table-2 and Figure-3.

In SNT, the pups under the double booster group showed the peak SN titer (GM: 3.83) at the 63^rd^ day post-primary vaccination, i.e., 21^st^ day after the second booster vaccination followed by a gradual decrease in antibody titer until the end of the study period. In contrast, the pups in the single booster group reached their peak SN titer (GM: 3.46) at the 42^nd^ day post-primary vaccination, i.e., 21^st^ day after the first booster vaccination which was slightly lower when compared to the double booster group. Although the difference in SN titers between the two groups of the pups was not statistically significant (p>0.05, i.e., 0.39), the pups under the double booster group maintained a better and higher immune response than the pups under the single booster group.

**Figure-4:**
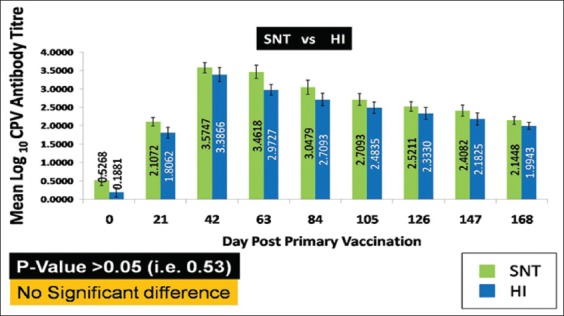
Comparison of the test results of hemagglutination inhibition and serum neutralization tests. The value indicates geometric mean log10 antibody titer ± standard error.

As the SN titers were found comparable with that of the HI titers, there was enough evidence to believe that the antibody titers of the vaccinated pups were protective in nature. Although there was no statistical significance (p>0.05, i.e., 0.53) between the test results of HI and SNT, SN titers were comparatively higher due to its higher sensitivity (Figure-4). Therefore, the commercially available CPV-2 based vaccine was found effective in providing protective immunity as indicated by HI and SNT. The results of SNT also indicated that the current vaccine strain (CPV-2) is capable of neutralizing the prevalent field strain (CPV-2a) in India, as a field CPV-2a strain was used for conducting SNT.

## Conclusion

In this study, serum samples were subjected to HI and SNT for measuring the CPV antibody titers from 27 healthy pups following primary and booster CPV vaccinations. The pups were categorized into two groups, the single booster and the double booster pups. The antibody titers in the double booster pups reached their peaks at the 63^rd^ day post-vaccination with GM of 3.57. The antibody titers in the single booster pups reached their peaks at the 42^nd^ day post-vaccination with a lower GM of 3.20. The double booster pups maintained a higher immune response than the single booster pups though the difference in titers was not statistically significant. SNT results indicated that the raised antibody titers were protective in nature though there was no statistical significance between HI assay and SNT. Therefore, the second booster vaccination may be useful in maintaining the protective titer for a prolonged period.

## Authors’ Contributions

JV, MVS, and HKM were involved in the design of this research work. JV performed the research. HKM monitored all the activities being a supervisor. MVS, PXA, VP, and JT assisted in this research work. MVS, JV, and HKM drafted and revised the manuscript. All authors read and approved the final manuscript.
